# Case Report: Hemodynamic challenges during the anesthetic management of a patient who presented with a cervical vagal schwannoma in southern Ethiopia: a rare clinical case

**DOI:** 10.3389/fsurg.2025.1569722

**Published:** 2025-06-24

**Authors:** Tajera Tageza Ilala, Gudeta Teku Ayano, Mengistu Yinges Kebede

**Affiliations:** Department of Anesthesia, College of Medicine and Health Sciences, Hawassa University, Hawassa, Ethiopia

**Keywords:** anesthesia, anesthesia for vagal schwannoma, case report, surgery, Schwann sheath, vagus schwannoma, vagus nerve

## Abstract

**Background:**

Vagal schwannoma is a benign tumor formed from the Schwann cells of the vagus nerve. Therefore, surgery is a viable treatment option for patients requiring tumor excision. However, anesthetic management of patients presenting with vagal schwannoma may pose a significant challenge to anesthetists because of an increased risk of intraoperative hemodynamic instability, cardiac arrhythmias, and postoperative functional impairment of the vocal cords, resulting in a life-threatening airway compromise.

**Case description:**

A 68-year-old female patient was referred to the otorhinolaryngology outpatient department of our institution with a history of lateral left neck swelling for 2 years, which started incidentally and gradually increased to attain the current size. CT of the neck revealed a schwannoma of the vagus nerve. General anesthesia was induced with propofol and succinylcholine following adequate preparation and premedication for surgical excision of the vagal schwannoma. She developed hemodynamic instability (a sudden decrease in her heart rate from 109 beats/min to 54 beats/min) and significant hypotension (her arterial blood pressure dropped from 168/87 mmHg to 78/36 mmHg) 30 min after the surgery. These symptoms were successfully treated with an intravenous injection of atropine sulfate (0.5 mg) and volume expanders, followed by vasopressor support (100 mcg intermittent bolus dose of epinephrine per 5 min twice), and the surgeon stopped the traction on the vagus nerve. The patient was awakened, extubated, and successfully transferred to the post-anesthesia care unit at the end of the procedure. No complications occurred during the post-operative period, and home discharge was made on day 4 of the post-operative period.

**Conclusion:**

Resection of a vagal schwannoma may pose significant challenges to the anesthetist during the intraoperative course due to the manipulation of the vagus nerve, mimicking the risk of hemodynamic instability. Hence, anesthetists should conduct adequate preoperative assessment and evaluation, be properly prepared, and closely monitor for and aggressively manage any hemodynamic instability to reduce morbidity and mortality during the resection of a vagus nerve schwannoma.

## Introduction

Schwannomas, or neurilemmomas, are benign encapsulated peri-neural tumors of neuro-ectodermal origin that arise from the Schwann cells in the brachial plexus of the nerve cell sheaths of the motor and sensory peripheral nerves (1). Schwannomas are rare benign tumors in the neck and head regions (2), and are often slow-growing, solitary, more-circumscribed, and confined (3). Approximately one-fourth to half of the vagus nerve tumors occurring outside the cranial space are found in the neck and head region (4), predominantly in the parapharyngeal space (5). Only one-third of schwannomas are found in the neck and head regions (6). Head and neck schwannomas commonly arise from the 8th cranial nerve, followed by the 9th, 11th, and 12th cranial nerves, and the sympathetic chain.

Except for the optic and olfactory nerves, all cranial, autonomous, and spinal nerves are shielded by a Schwann sheath (2, 5). Cervical vagal schwannomas are extremely rare (7). Vagal tumors in the brachial plexus are unusual nerve sheath tumors that may arise from the peripheral, cranial, or autonomic nerves of the body, excluding optic and olfactory nerves (8). Hence, given the rarity of cervical vagal tumors, they require careful diagnosis using imaging modalities to differentiate them from other neck masses (9, 10). This tumor commonly occurs in patients 30–50 years of age (11, 12), with no sex or race preponderance (13, 14). Most patients present with an asymptomatic neck mass, which may manifest as a cough on mass palpation (15).

An early diagnosis and intervention may be challenging preoperatively due to the asymptomatic nature of tumor presentation (8). Schwannomas always pose a diagnostic challenge because of their non-specific clinical signs and slow growth that manifests with subtle symptoms, making their diagnosis challenging. Most often, the diagnosis is made after a histopathological examination (2, 16, 17). Preoperative imaging provides crucial information related to the tumor’s location, extent, and origin (12, 16).

Surgery is the primary management strategy of choice for schwannomas. The surgery plan varies considerably to enable the full removal of the schwannoma’s capsule to avoid tumor regrowth (2, 18, 19). The close proximity of the vagus nerve to great vessels, including the carotid artery and jugular veins, may cause technical difficulties during surgery, which may increase the risk of significant bleeding from unintentional vascular injury or mechanical compression during surgical dissection (11). There is also a high risk of intraoperative cardiac dysrhythmias, hemodynamic instability, and postoperative complications resulting from vagal stimulation, vessel compression, or nerve trauma (20, 21).

The provision of anesthesia for patients presenting with vagal nerve schwannomas may pose a significant challenge to anesthesia providers because manipulation of the tumor may lead to unstable blood pressure, severe bradycardia, and even cardiovascular collapse. Hence, close monitoring and management of cardiac arrhythmias and hypotension and an attempt to minimize vagal overdominance should be undertaken to prevent and manage the hemodynamic perturbations that occur during the intraoperative period (18, 20, 22). Postoperative airway obstruction may result from vagus nerve injury during surgery, which may require careful airway examination during the early postoperative period to exclude vocal cord palsy (23, 24).

We present the unique hemodynamic challenges that arose during the intraoperative anesthetic management of a patient who presented with a cervical vagal schwannoma in southern Ethiopia.

## Case description

A 68-year-old female patient was transferred to the otorhinolaryngology surgical department of our institution with a history of lateral left neck swelling for 2 years, which started incidentally and gradually increased to attain the current size. She had a history of tinnitus, left ear pain, left ear pruritus, burning sensation, cheesy white discharge from the left ear, and an initial partial headache, which later evolved into a global headache. She had a history of subtotal thyroidectomy 6 years prior and had no documented anesthesia or surgical complications. The physical assessment showed a non-tender, soft, non-fixed, smooth-surfaced mass on the left neck. The patient’s cervical lymph nodes were not palpable. During the preoperative evaluation, the patient was stable, and her non-invasive blood pressure was 112/87 mmHg, her heart rate was 91 beats/min and regular and full in volume, and her axillary temperature was 36.7°C. The patient’s complete blood count results were normal. The electrocardiography (ECG) and chest radiography results were normal. Echocardiographic findings revealed normal cardiac function with an ejection fraction of 65%.

A computed tomography scan of the neck revealed a heterogeneous mass of neck soft tissue measuring 1.8 cm  × 1.6 cm  × 2.6 cm [axial Antero-Posterior (AP) and longitudinal dimension] in the left carotid space with a heterogeneous lesion-enhanced hyper-dense soft tissue mass displacing the left internal carotid artery and left internal jugular vein anteromedially, suggestive of a nerve sheath tumor (vagal schwannoma).

Following careful investigation and preparation, tumor removal surgery was conducted under general anesthesia. The night before the operation, the risks and benefits of the procedure were openly discussed with the patient and her family, and written informed consent was provided. A plastic surgeon was consulted to perform the neurovascular repair procedure on the nerve. An intensive care unit bed was prepared to treat any postoperative complications resulting from vagus stimulation or exacerbation.

**Figure 1:**
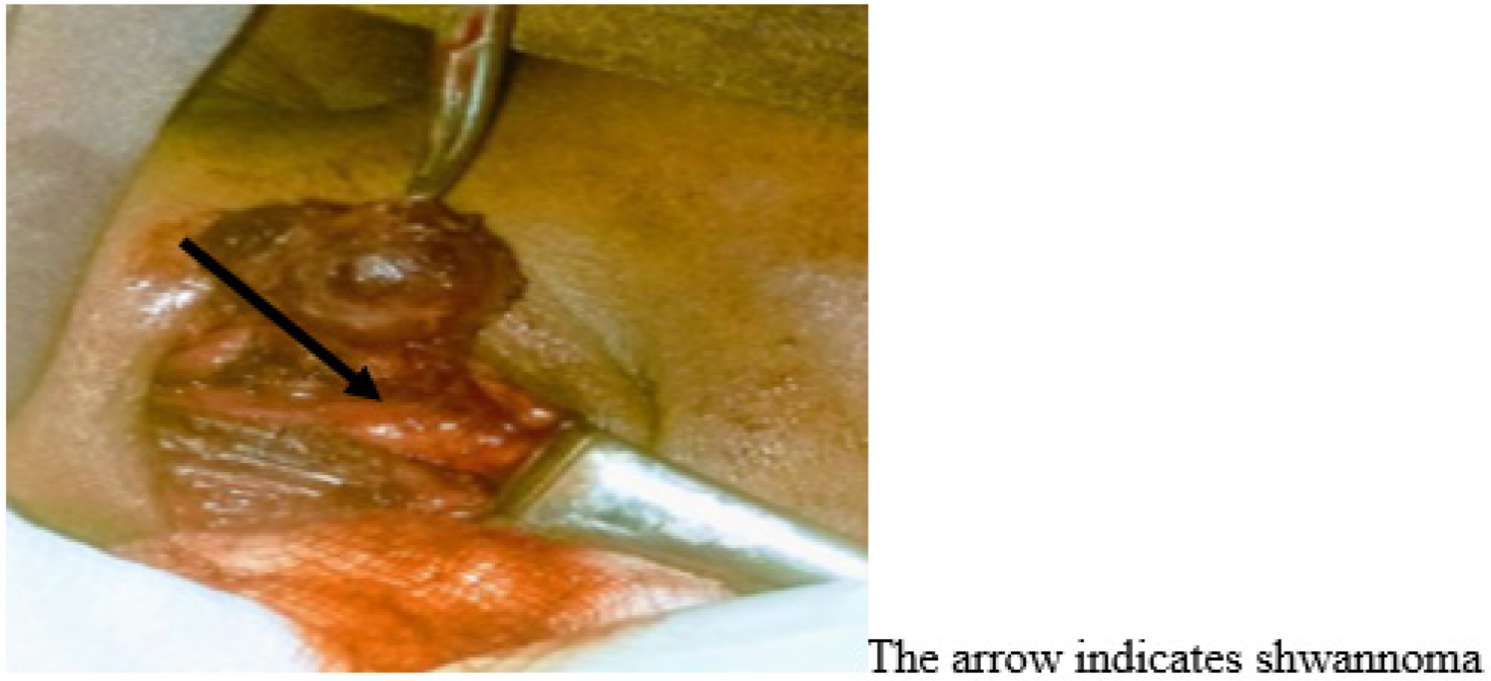
Resection of a cervical vagal schwannoma in a 68-year-old female patient.

On arrival at the operating room, bilateral 18G intravenous (IV) cannulae were established following placement of the standard monitors (pulse oximetry, non-invasive blood pressure, five-lead electrocardiography, and temperature). A defibrillator was then prepared for possible cardiac resuscitation. Baseline vital signs were as follows: heart rate, 106 beats/min; arterial blood pressure, 124/84 mmHg; and temperature, 37°C. Prior to the induction of general anesthesia, we premedicated the patient with IV morphine (6 mg), dexamethasone (8 mg), and atropine (0.5 mg). General anesthesia was induced using ketofol (1:2) with suxamethonium chloride (2 mg/kg). Direct rigid laryngoscopy was used to intubate the trachea with a 6.5 cm endotracheal tube. Isoflurane (1%–2%) with 100% oxygen was used for maintenance of anesthesia, and muscle relaxation was maintained with vecuronium. The induction of general anesthesia and tracheal intubation was uneventful. The patient’s end-tidal CO_2_ (EtCO_2_) was closely monitored to detect tubing disconnections, apnea, leaks, and adequate ventilation. Surgery was initiated following the induction of general anesthesia after the airway devices were deemed properly placed and safely secured. The incision was placed in an upper neck skin crease extending from the mastoid process toward the ischial tubercle, overlying the mass. The skin incision was deepened through the platysma to expose the anterior border of the sternocleidomastoid muscle. The fascia anterior to the sternocleidomastoid muscle was incised and the sternocleidomastoid muscle was retracted laterally to expose the mass. The carotid sheath was dissected and the internal jugular vein, carotid artery, and vagus nerve were exposed. The tumor arose from the vagus nerve, which separates the internal jugular vein from the external carotid artery. The entire tumor was safely separated from the nerve and completely excised, while preserving the anatomical continuity and functionality of the nerve (Figure 1).

During the intraoperative period, 30 min after the start of the procedure, the patient developed bradycardia (a sudden decrease in the heart rate from 109 beats/min to 54 beats/min) and significant hypotension (her arterial blood pressure dropped from 168/87 mmHg to 78/36 mmHg) due to traction on the vagus nerve and mass dissection from the nearby structure by the surgeon. We immediately injected atropine sulfate (0.5 mg) and noticed that the surgeon had stopped the traction on the vagus nerve. The patient’s hypotension was successfully managed with a rapid infusion of volume expanders (20 ml/kg of 0.9% normal saline), followed by vasopressor support (100 µg intermittent bolus dose of epinephrine twice every 5 min). The patient’s heart rate increased to 70 beats/min, and normal blood pressure was restored 10 min after resuscitation. The surgeon resumed the procedure after achieving adequate hemodynamic parameters. The total durations of surgery and anesthesia were 2 h and 2 h and 30 min, respectively. Moreover, the patient’s neuromuscular blockade was reversed and her vocal cords were checked under direct laryngoscopy for possible vagus nerve injuries. The patient was awakened, extubated, and successfully transferred to the post-anesthesia care unit at the end of the procedure. Standard monitoring devices were attached upon the patient’s immediate post-anesthesia care unit admission. Oxygen supplementation was provided through nasal prongs at 2 L/min for the first 15 min to support oxygenation and ventilation. Close monitoring and follow-up were performed for the first 6 h in the recovery room, and the patient was then transferred to the Ear, Nose, and Throat (ENT) ward after adequate discharge criteria were met. The postoperative course was uneventful and no complications were noted. Home discharge was made on day 4 of the postoperative course and the patient completely recovered. The histological test results confirmed a vagal schwannoma with ganglion cells and nerve fibers.

## Discussion

A vagal Schwannoma is an unusual benign lesion that develops in the soft tissues of the neck and head region and primarily affects the vagus nerve sheath. Affected patients may present with subtle clinical symptoms such as a slow-growing, non-tender neck swelling, ear complaints, and a cough. In addition to their rare occurrence, cervical vagal tumors may pose a greater hemodynamic challenge to the anesthesia provider during the intraoperative period (6, 21, 22, 25, 26).

Intraoperative anesthetic care may present greater challenge due to the traction on the vagus nerve, and surgical manipulation may increase the risk of vagal stimulation, resulting in hemodynamic instability such as severe bradycardia, precipitated hypotension, aberrant ECG readings, and even cardiac arrest. Similarly, our patient developed sudden bradycardia and hypotension during the intraoperative period due to surgical traction and tumor manipulation. These symptoms were aggressively managed with intravenous fluid, vasopressors, and cessation of surgical stimulation (20, 26).

Ansari et al. reported the incidence of intraoperative cardiac arrest during the surgical excision of a large vagal schwannoma that may have occurred due to the direct stimulation of the vagus nerve, causing depression of the SA nodal rhythm and cardiac conduction systems and precipitating cardiac arrest. The patient in our case did not experience cardiac arrest. This may be related to the early diagnosis and aggressive management in our case, which may also have contributed to the lower severity of the condition (4, 18).

The localized effects of the mass or tumor may progressively compress the vocal cords, causing hoarseness and airway obstruction preoperatively. Approximately 12% of patients who present with a vagal schwannoma in the neck region experience vocal cord paralysis (23). However, in our case, the patient had no preoperative airway manifestations.

Surgical excision of a large vagal schwannoma can be associated with significant postoperative airway obstruction, which may manifest as hoarseness, stridor, and complete airway obstruction resulting from vocal cord paralysis (24, 26). Similarly, Singh (6) and Ali et al. (22) reported that approximately 85% of patients developed vocal cord palsy following the surgical excision of a vagal schwannoma due to nerve damage or edema. However, our patient did not develop vocal cord disorders. Hence, it is crucial to examine vocal cord mobility before and immediately after the procedure to assess the possibility of neural injury. We performed a detailed preoperative evaluation to assess vocal cord function and performed a laryngoscopic examination during the immediate postoperative period, as the normal functioning of the vocal cords was confirmed prior to extubation. Furthermore, anesthesia providers should perform a careful airway evaluation and closely monitor the postoperative course to ensure the early recognition, diagnosis, and initiation of proper management of any associated ventilation problems.

Finally, the results of this study may help in hypothesis generation in the future, rather than provide a conclusion, as this study only includes the data from a single patient.

## Conclusion

A cervical Schwann-cell tumor of the vagus nerve is a rare benign tumor that affects the vagus nerve sheath. Appropriate preoperative radiographic workups, such as CT scans and magnetic resonance imaging (MRI), were carefully performed to ensure early diagnosis and management. The mechanical effects of the mass on the great vessels and nerves necessitate the surgical resection of the mass. Resection of a vagal schwannoma may pose significant challenges to the anesthetist during the intraoperative course owing to the manipulation of the vagus nerve during surgery, increasing the risk of hemodynamic instability, which can even progress to cardiovascular collapse. Hence, the anesthetist should conduct an adequate preoperative assessment and evaluation, prepare properly (be ready for immediate cardiac resuscitation), perform close intraoperative monitoring, ensure good communication, and aggressively manage any hemodynamic instability, which may reduce the morbidity and mortality associated with schwannoma resection and vagus nerve manipulation. Intraoperative hemodynamic instability was successfully managed after traction on the vagus nerve and tumor manipulation in a 68-year-old female patient during resection of a cervical vagal schwannoma.

## Data Availability

The original contributions presented in the study are included in the article/Supplementary Material, further inquiries can be directed to the corresponding author.
